# Effects of Bacillus Calmette-Guérin (BCG) vaccination at birth on T and B lymphocyte subsets: Results from a clinical randomized trial

**DOI:** 10.1038/s41598-017-11601-6

**Published:** 2017-09-29

**Authors:** Nina Marie Birk, Thomas Nørrelykke Nissen, Jesper Kjærgaard, Hans Jacob Hartling, Lisbeth Marianne Thøstesen, Poul-Erik Kofoed, Lone Graff Stensballe, Andreas Andersen, Ole Pryds, Mihai G. Netea, Christine Stabell Benn, Susanne Dam Nielsen, Dorthe Lisbeth Jeppesen

**Affiliations:** 1 Department of Pediatrics, Copenhagen University Hospital, Hvidovre, Kettegårds Allé 30, 2650 Hvidovre, Denmark; 2 The Department of Pediatrics and Adolescent Medicine, Copenhagen University Hospital, Rigshospitalet, Blegdamsvej 9, 2100 Copenhagen, Denmark; 3 Department of Infectious diseases, Copenhagen University Hospital, Rigshospitalet, Blegdamsvej 9, 2100 Copenhagen, Denmark; 4 Department of Pediatrics, Kolding Hospital, Sygehusvej 24, 6000 Kolding, Denmark; 5 Research Center for Vitamins and Vaccines (CVIVA), Bandim Health Project, Statens Serum Institut, Artillerivej 5, 2300 Copenhagen, Denmark; 6 Department of Internal Medicine and Radboud Center for Infectious Diseases, Radboud University Medical Center, Geert Grooteplein 8, 6525 GA Nijmegen, The Netherlands

## Abstract

The Bacillus Calmette–Guérin vaccine (BCG) has been associated with beneficial non-specific effects (NSEs) on infant health. Within a randomized trial on the effect of neonatal BCG on overall health, we investigated the possible immunological impact of neonatal BCG vaccination on lymphocyte subsets, determined by flow cytometry. In 118 infants blood samples were obtained 4 (±2) days post randomization to BCG vaccination or no intervention, and at 3 and 13 months of age. No effects of BCG were found at 4 days. However, BCG increased proportions of effector memory cells at 3 months (Geometric mean ratio (GMR) 1.62, 95% confidence interval (CI) (1.20–2.21), p = 0.002 for CD4^+^ T cells and GMR 1.69, 95% CI (1.06–2.70), p = 0.03 for CD8^+^ T cells), and reduced proportions of late differentiated CD4^+^ T cells (GMR = 0.62, 95% CI (0.38–1.00), p = 0.05) and apoptotic CD4^+^ T cells at 13 months (GMR = 0.55, 95% CI (0.32–0.92), p = 0.03). In conclusion, limited overall impact of neonatal BCG vaccination on lymphocyte subsets was found in healthy Danish infants within the first 13 months of life. This is in line with the limited clinical effects of BCG observed in our setting.

## Introduction

The Bacillus Calmette-Guérin vaccine (BCG) used against tuberculosis (TB) has been administered to billions of infants and is still one of the most used vaccines worldwide^1^. The observation that BCG may have beneficial non-specific clinical effects (NSEs) beyond the specific protection against TB dates back to the 1930s^2^, and today BCG is used in standard treatment of bladder cancer.

Both observational studies^3–6^ and recent randomized clinical trials^7,8^ from low-income countries found that neonatal BCG vaccination reduced all-cause morbidity and mortality from infections other than TB. In 2014, the Strategic Advisory Group of Experts (SAGE) on Immunization conducted a review of the NSEs of BCG and concluded that BCG was associated with reductions of overall mortality, that was not explained by prevention of TB; recommending further research into the NSEs of BCG^9^.

The immunological mechanisms underlying the potential NSEs of BCG were also reviewed by SAGE; the available studies were too heterogeneous to provide conclusive evidence^10^. It has been proposed that cross-protection mediated by heterologous T cell memory activation could be one of the underlying mechanisms^11^. Furthermore, recently BCG was found to induce epigenetic reprogramming of monocytes in adults, causing increased cytokine release in response to nonrelated pathogens, or so-called ‘trained innate immunity’^12,13^. Finally, BCG was found to induce a nonspecific response to non-mycobacterial infections mediated by heterologous Th1/Th17 responses^14^, representing yet another potential biological mechanism which could explain NSEs.

Within a randomized clinical trial of neonatal BCG in Danish children, we aimed to evaluate effects of BCG within the adaptive immune system by assessing the distribution of T and B lymphocyte subsets in peripheral blood 4 days post-randomization, and 3, and 13 months of age

## Materials and Methods

### Setting

The present study was nested within The Danish Calmette Study, a multicenter, randomized clinical trial with 1:1 allocation of newborn infants to receive either BCG vaccination or no intervention within 7 days of birth^15^. Randomization was done using a centralized on-line system with stratification according to GA of the child (<37 weeks vs. ≥37 weeks). The allocation sequence was computer generated in permuting blocks of 2:4:6. Between October 2012 and December 2013 a total number of 4,262 newborns were randomized. The inclusion criteria were gestational age ≥32 weeks and a birth weight ≥1000 grams. Exclusion criteria were maternal intake of immune modulating medicine during pregnancy or signs of severe illness, or major malformation in the newborn. Immediately following randomization the intervention group was vaccinated intra-dermally with BCG vaccine, SSI^©^ strain 1331, in the standard dose of 0.05 ml in the upper, lateral part of the left shoulder. Design and methods have been described in detail elsewhere^16^. No placebo was used, since it is not possible to mimic the pustule at the infants arm following a BCG vaccination, hence parents were not blinded to the intervention. However, the study staff was blind to the intervention at data collection by covering the site of a potential scar at the clinical examinations by a plaster and blood samples were given anonymized study id numbers.

### Inclusion into the present study

**Figure 1:**
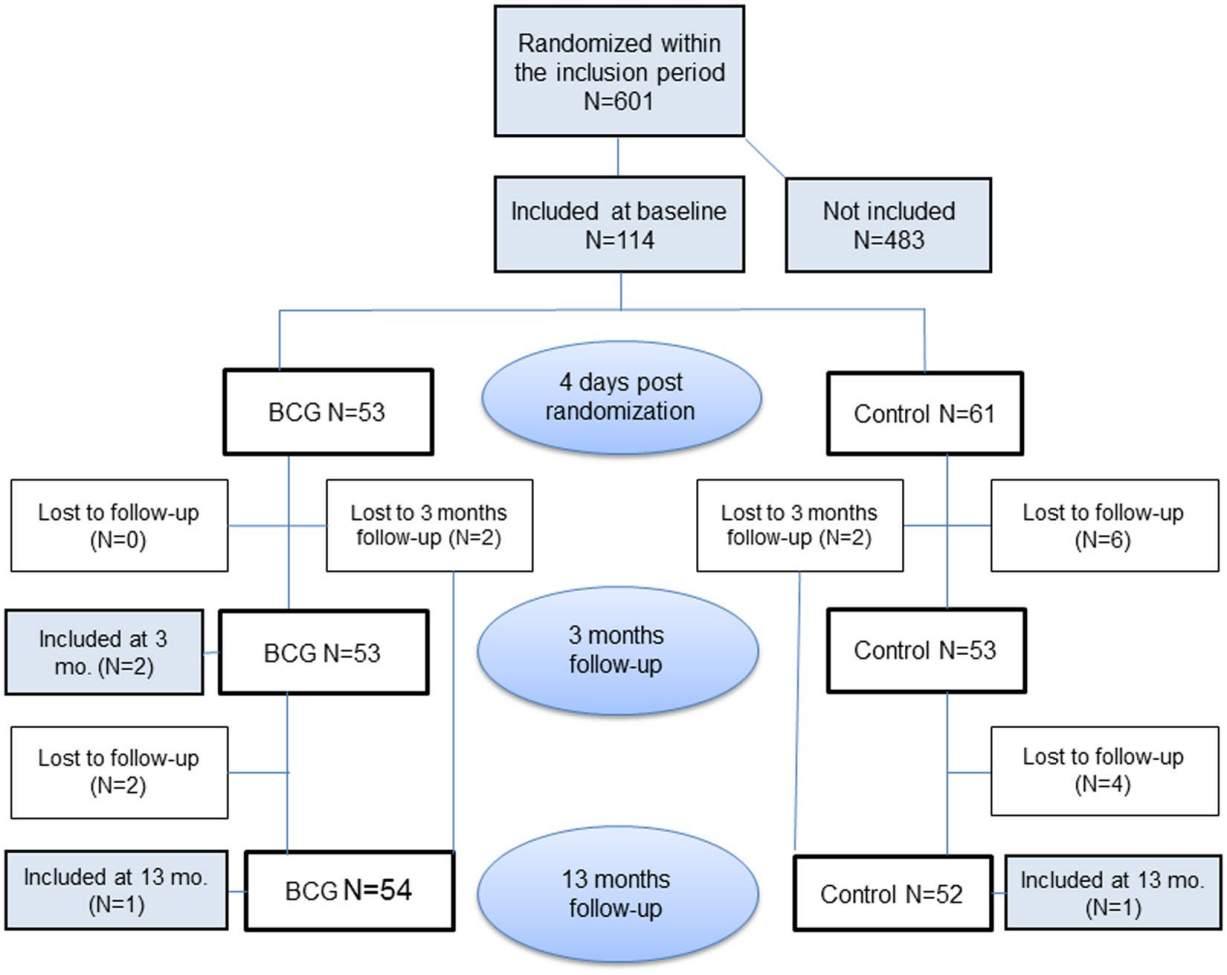
Flowchart of infants included into the study assessing the effect of BCG on T and B cell subsets by flow cytometry. A total of 601 infants were randomized within the Danish Calmette study in the inclusion period of the present substudy. Overall 118 infants participated in the present study; 114 infants were enrolled at day 4, further 2 infants were enrolled at 3 and 13 months.

Parents giving birth at Copenhagen University Hospital, Hvidovre, who had already provided consent for participating in the Danish Calmette Study within the inclusion period from June 2013 to December 2013, were prior to randomization invited to participate in this immunological substudy (Fig. 1). Infants, who were randomized on days which were compatible with later blood sampling, were invited to participate. Unfortunately, we collected no data on refusals.

### Background information and clinical follow-up

Background information was collected by a structured telephone interview conducted in the third trimester of pregnancy. At 3 and 13 months of age all infants were invited for a clinical examination at the hospital preceded by a follow up telephone interview.

### Blood samples and flow cytometry

To assess T and B cell subsets, blood samples were collected at three time points: at 4 (±2) days post randomization, and at 3 months and 13 months of age.

**Figure 2:**
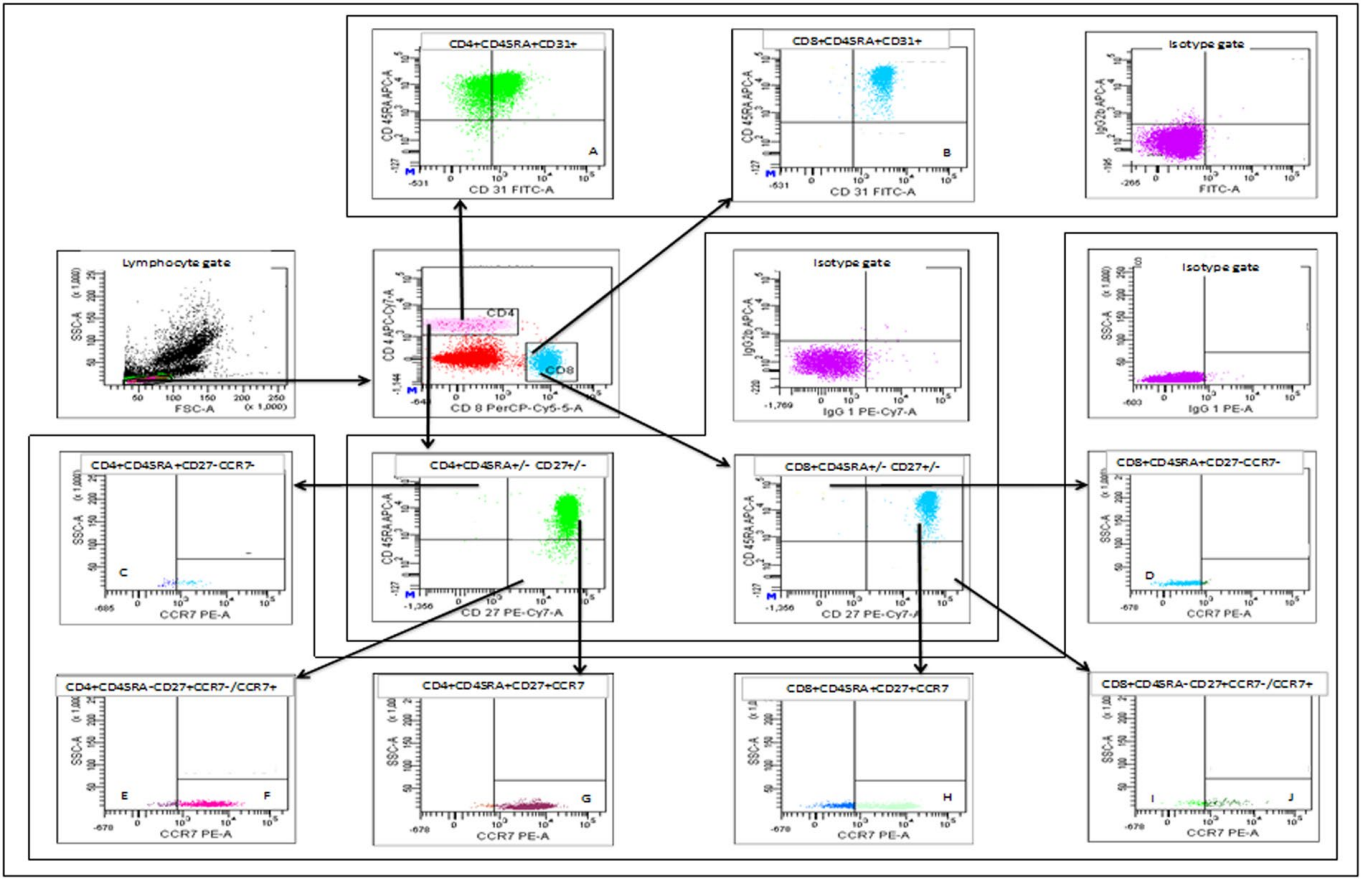
Gating strategy. Representative plots illustrating gating strategies within the CD4+ and CD8+ T cell compartment of (**A**, **B**) **Recent thymic emigrants** (CD45RA+CD31+), (**C**, **D**) **Late differentiated cells** (CD45RA+CD27-CCR7-), (**E**, **I**) **Effector memory cells** (CD45RA-CD27+CCR7-), (**F**, **J**) **Central memory cells** (CD45RA-CD27+CCR7+) (**G**, **H**) **Naïve cells** (CD45RA+CD27+CCR7+) including relevant **isotype control**.

**Figure 3:**
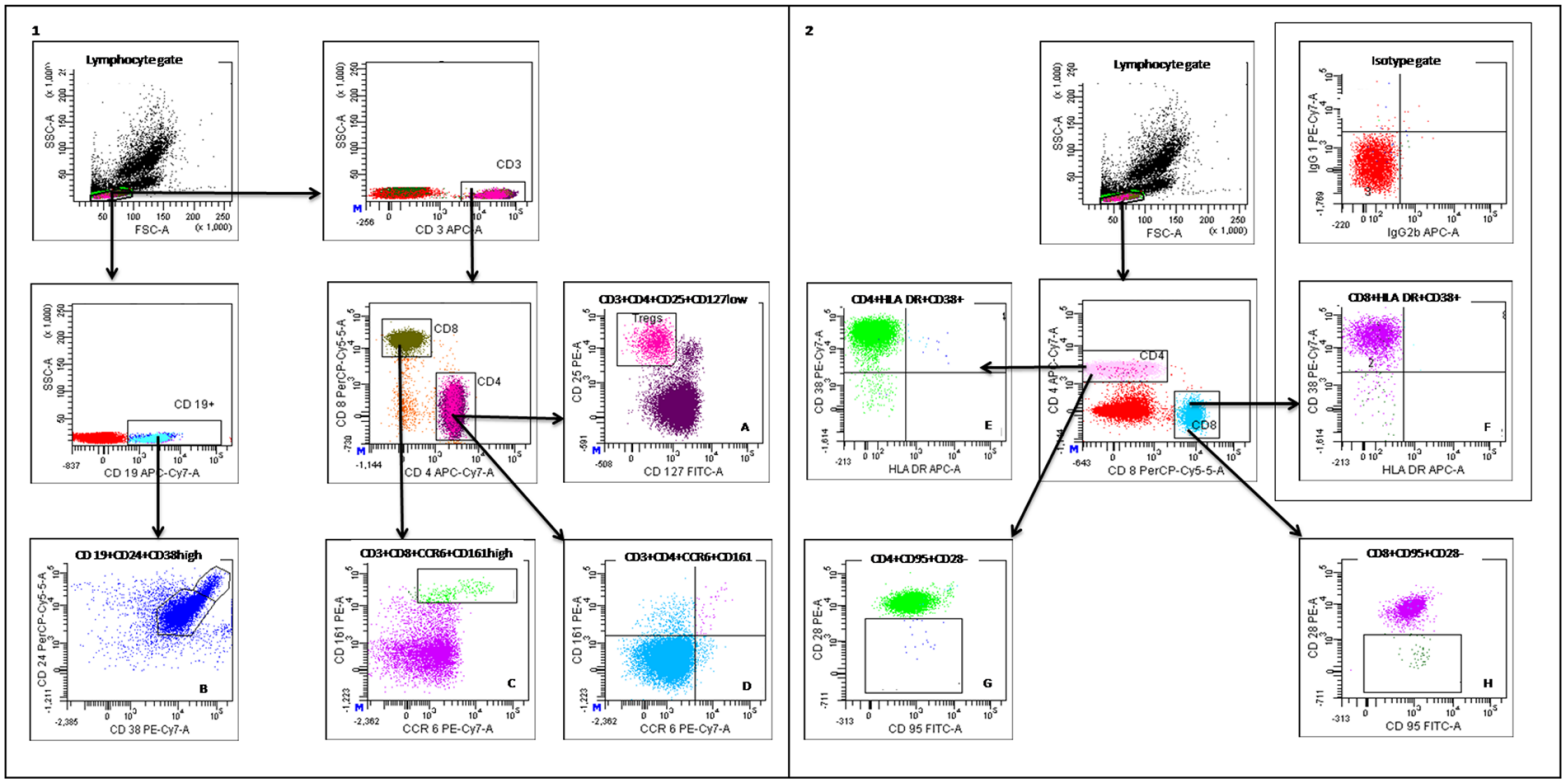
Gating strategy of phenotypes of T and B cells assessed in infants randomized to BCG or no BCG at birth. Representative plots illustrating gating strategies within the CD19 + B cell compartment (1) and CD4+ and CD8+ T cell compartments (1 and 2) of (**A**) **T regulatory cells** (CD3 + CD4 + CD25 + CD127low), (**B**) **CD19 + CD24** ^**high**^ **+ CD38** ^**high**^, (**C**) **TC 17 cells** (CD3 + CD8 + CCR6 + CD161high), (**D**) **TH 17 cells** (CD3+CD4 + CCR6 + CD161+), (**E,F**) **Chronically activated cells** (HLADR + CD38+), (**G,H**) **Apoptotic cells** (CD95 + CD28−), including relevant isotype control.

Flow cytometry to determine lymphocyte subsets was done using peripheral blood collected in 2 mL EDTA tubes. Gating strategies are shown in Figs 2 and 3. In brief, 100 µL of EDTA blood was incubated with fluorescent dye–conjugated monoclonal antibodies at room temperature for 20 minutes according to the manufacturer’s instructions. Erythrocytes were then lysed with 2 ml of Lysing Solution (Becton Dickinson (BD), Franklin Lakes, NJ, USA) at room temperature for 20 minutes, where after the samples were washed and re-suspended in FACS Flow (BD). Monoclonal antibodies used were 1-phycoerythrin (PE), peridinin chlorophyll proteins–cyanine (PerCP-Cy5.5), 1-fluorescein isothiocyanate (FITC), 1-allophycocyanin (APC), 1-phycoerythrin combined with cyanine dye Cy7 (PE-CY7), and 1-allophycocyanin combined with an analog of Cy7 (APC-H7) for isotype controls. To determine specific cell subsets CD28-PE, CCR7-PE, CD25-PE, CD161-PE, CD8-PerCPCy5.5, CD24- PerCPCy5.5, CD95-FITCH, CD31- FITCH, CD127- FITCH, HLADR-APC, CD45RA- APC, CD3- APC, CD27-PE-Cy7, CCR6-PE-Cy7, CD38- PE-Cy7, CD4-APC-H7, and CD19-APC-H7 were used, all purchased from BD.

Acquisition was done using a 6-color FACS Canto (BD), and data were processed using FACS Diva software (BD). A minimum of 100,000 cells were acquired in each sample. All lymphocyte gating was conducted by one examiner (NMB) blinded to the intervention group. Lymphocyte subsets are given as proportions (%)of the parent cell population concerned (CD4+ T cells, CD8+ T cells, or CD19+ B cells) as well as absolute cell counts (cells/µl) (Table 1). Geometric means are given in Supplementary Table A. Absolute cell counts were calculated by multiplying proportion of cells by total lymphocyte count (cells/µl) obtained from a differential count (Sysmex XE-5000, Sysmex, Mundelein, IL, USA) analyzed at the Department of Clinical Biochemistry, Copenhagen University Hospital, Hvidovre.

**Table 1:** Overall effects of neonatal BCG on lymphocyte subsets: **immature cells and thymic output** (Naïve T cells and recent thymic emigrants (RTE)), **immune regulation** (T regulatory cells (Tregs), CD19+CD24high+CD38high, Th 17 cells, Tc 17 cells), and **T cell homeostasis** (central memory T cells, effector memory T cells, late differentiated T cells, chronic activated cells and Apoptotic cells) assessed by flow cytometry.

	**4 days**						**3 months**						**13 months**					
	Proportions (%)			Absolute cell counts (cells/µL)			Proportions (%)			Absolute cell counts (cells/µL)			Proportions (%)			Absolute cell counts (cells/µL)		
	GMR^a^ (95% CI)			GMR (95% CI)			GMR (95% CI)			GMR (95% CI)			GMR (95% CI)			GMR (95% CI)		
	BCG vs. no BCG		p value	BCG vs. no BCG		p value	BCG vs. no BCG		p value	BCG vs. no BCG		p value	BCG vs. no BCG		p value	BCG vs. no BCG		p value
**CD 4+ T cell subsets**																		
*CD4+ T cells*				1.18	(0.94–1.49)	0.16				1.01	(0.89–1.14)	0.91				0.94	(0.78–1.13)	0.49
Naive cells	0.99	(0.98–1.02)	0.99	1.18	(0.93–1.50)	0.18	0.95	(0.97–0.99)	**0.01**	0.99	(0.87–1.13)	0.89	0.99	(0.97–1.01)	0.16	0.94	(0.78–1.13)	0.51
Recent thymic emmigrants	1.03	(0.96–1.11)	0.40	1.21	(0.94–1.55)	0.13	1.03	(0.97–1.08)	0.37	1.04	(0.90–1.19)	0.61	1.01	(0.95–1.07)	0.85	0.96	(0.79–1.17)	0.68
Tregs	1.05	(0.92–1.20)	0.46	1.28	(0.97–1.67)	0.08	1.00	(0.93–1.09)	0.92	0.93	(0.73–1.18)	0.53	1.04	(0.96–1.12)	0.59	0.98	(0.83–1.16)	0.84
Th 17 cells	1.17	(0.91–1.50)	0.22	1.30	(0.92–1.82)	0.13	1.13	(0.98–1.31)	0.10	1.02	(0.81–1.29)	0.84	1.05	(0.92–1.20)	0.49	0.99	(0.84–1.17)	0.92
Central memory cells	1.14	(0.75–1.73)	0.53	1.29	(0.80–2.07)	0.29	1.22	(0.84–1.77)	0.30	1.24	(0.85–1.83)	0.28	1.10	(0.96–1.85)	0.09	1.26	(0.89–1.78)	0.19
Effector memory cells	1.23	(0.85–1.77)	0.27	1.35	(0.90–2.02)	0.15	1.62	(1.20–2.21)	**0.002**	1.64	(1.21–2.23)	**0.002**	0.91	(0.70–1.20)	0.51	0.87	(0.67–1.13)	0.29
Late differentiated cells	1.01	(0.74–1.38)	0.95	1.18	(0.85–1.67)	0.31^c^	1.06	(0.74–1.52)	0.76	1.06	(0.74–1.52)	0.77	0.62	(0.38–1.00)	**0.05**	0.59	(0.38–0.93)	**0.02**
Chronic activated cells	1.16	(0.91–1.48)	0.23	1.38	(0.97–1.95)	0.07	0.86	(0.63–1.17)	0.33	0.85	(0.62–1.18)	0.33	0.86	(0.61–1.20)	0.37	0.85	(0.62–1.18)	0.33
Apoptotic cells	1.03	(0.82–1.30)	0.80	1.21	(0.87–1.68)	0.25	0.89	(0.68–1.18)	0.42	0.89	(0.68–1.16)	0.39	0.55	(0.32–0.92)	**0.03**	0.53	(0.31–0.91)	**0.02**
**CD8+ T cell subsets**																		
*CD8+ T cells*				1.08	(0.83–1.42)	0.55				0.90	(0.77–1.06)	0.21				0.89	(0.74–1.07)	0.21
Naive cells	0.99	(0.97–1.01)	0.29	1.06	(0.80–1.39)	0.70	0.98	(0.90–1.06)	0.56	0.88	(0.76–1.03)	0.11	1.06	(0.97–1.16)	0.18	0.93	(0.77–1.12)	0.45
Recent thymic emmigrants	0.99	(0.98–1.00)	0.36	1.06	(0.81–1.39)	0.68	1.00	(0.95–1.05)	0.95	0.91	(0.78–1.05)	0.20	1.03	(0.98–1.09)	0.23	0.91	(0.76–1.08)	0.28
Tc 17 cells	0.93	(0.74–1.17)	0.56	0.93	(0.67–1.29)	0.67	0.91	(0.74–1.13)	0.40	0.73	(0.50–1.07)	0.11	1.03	(0.82–1.28)	0.81	0.93	(0.74–1.18)	0.54
Central memory cells	0.94	(0.70–1.27)	0.67	0.97	(0.67–1.40)	0.86	1.15	(0.87–1.53)	0.33	1.00	(0.71–1.42)	0.99	1.30	(0.94–1.81)	0.11	1.11	(0.75–1.64)	0.59
Effector memory cells	0.98	(0.66–1.45)	0.91	0.99	(0.61–1.59)	0.95	1.69	(1.06–2.70)	**0.03**	1.41	(0.83–2.41)	0.20	0.99	(0.68–1.45)	0.97	0.85	(0.56–1.29)	0.43
Late differentiated cells	0.91	(0.62–1.34)	0.63	0.91	(0.58–1.43)	0.69	0.96	(0.55–1.70)	0.90	0.84	(0.45–1.55)	0.57	0.59	(0.29–1.18)	0.14	0.53	(0.25–1.11)	0.09
Chronic activated cells	1.07	(0.78–1.48)	0.66	1.25	(0.81–1.92)	0.32	0.82	(0.57–1.17)	0.27	0.72	(0.47–1.12)	0.14	0.90	(0.66–1.21)	0.48	0.80	(0.56–1.11)	0.17
Apoptotic cells	1.02	(0.82–1.26)	0.88	1.06	(0.76–1.48)	0.71	0.94	(0.65–1.37)	0.76	0.84	(0.54–1.32)	0.46	0.83	(0.61–1.12)	0.22	0.75	(0.52–1.09)	0.13
**CD 19+ B cell subsets**																		
*CD19+ B cells*				1.08	(0.84–1.41)	0.54				1.02	(0.87–1.19)	0.84				1.00	(0.84–1.19)	0.99
CD19+CD24^high^+CD38^high^	0.99	(0.96–1.02)	0.52	1.08	(0.83–1.42)	0.56	0.98	(0.86–1.12)	0.78	0.99	(0.81–1.20)	0.89	1.05	(0.93–1.19)	0.45	1.04	(0.84–1.28)	0.74

^a^Geometric mean ratio

### Outcomes

The predefined primary outcome was proportions of T and B lymphocyte subsets 4 days post randomization. The secondary outcomes were the cell subsets measured as absolute counts at 4 (±2) days post randomization, and 3 and 13 months and as proportions at 3 and 13 months. The cell subsets were grouped into the following categories:

#### Immature T cells and thymic output

Recent thymic emigrants (RTE) ((CD4^+^/CD8^+^) CD45RA^+^CD31^+^) and Naive cells ((CD4^+^/CD8^+^) CD45RA^+^CD27^+^CCR7^+^)^17,18^.

#### Immune regulation

Regulatory T cells (Tregs) (CD3^+^CD4^+^CD25^+^CD127^low^)^19^, B cells with the phenotype of CD19^+^CD24^high^CD38^high^ ^20^, Th17 cells (CD3+CD4^+^CCR6^+^CD161^+^)^21,22^, and Tc17 cells (CD3^+^CD8^+^CCR6^+^CD161^high^)^23,24^.

The B cell phenotype CD19^+^CD24^high^CD38^high^ has been shown to be enriched for IL-10 producing cells and to have regulatory capacity^20^. Due to the lack of IL-10 measurements in this study, this cell type will be referred to by its phenotype.

#### T cell homeostasis

Central memory cells ((CD4^+^/CD8^+^) CD45RA^−^CD27^+^CCR7^+^), Effector memory cells ((CD4^+^/CD8^+^) (CD45RA^−^CD27^+^CCR7^−^), Late differentiated cells ((CD4^+^/CD8^+^) CD45RA^+^CD27^−^CCR7^−^), Chronic activated cells ((CD4^+^/CD8^+^) HLADR^+^CD38^+^), and Apoptotic cells ((CD4^+^/CD8^+^) CD95^+^CD28^−^)^18^.

### Sample size

The sample size was based on previous work with T cell subsets where the mean of recent thymic emigrants (RTE) in adults was 24.1 (SD 10.1). To detect a difference of 30% between groups, we needed to include 43 in each group (2-sided test, power 90%, alpha 0.05). To account for dropouts, we aimed to include a minimum of 100 infants.

### Statistical methods

As data were not consistently log-normally distributed, bootstrapped sampling with confidence intervals as the 2.5% and 97.5% percentiles was conducted, but showed no difference from the standard normal-based confidence interval (CI); hence the latter will be shown. All infants in this substudy followed allocation.

The primary estimate of the BCG effect is presented as the geometric mean ratio (GMR) with 95% CI obtained as the anti-logged coefficient from a linear regression with the log-concentration or log-proportion as outcome and randomization group as covariate.

On the basis of previous findings of BCG having more pronounced beneficial non-specific effects in girls^25^ we pre-specified that primary outcome estimates would be assessed both overall and stratified by sex.

The following predefined potential effect modifiers were assessed: age at randomization (which was specified as 0–1 day versus 2–7 days, since most children were included during the first days of life and further grouping would result in small group sizes and limited power) and maternal BCG (yes/no).

To assess changes in each cell subpopulation over time (4 days, 3 months, 13 months) the hypothesis of equal BCG-effect over time (no time-interaction) was tested in a model with BCG, time, and the interaction BCG*time, leaving the correlation structure unspecified.

To test a potential effect of BCG within groups of subsets according to their function: “Immature T cells and thymic output”, “Immune regulation”, and “T cell homeostasis” post-hoc analysis comparing total effect within each group were performed. We accounted for potential correlation between the cell subsets by using the clustered variance estimator.

Since we have no data on which infants that were invited into this substudy but refused to participate, a comparison was made between baseline characteristics in infants included into the present study and infants included at Copenhagen University Hospital, Hvidovre in the inclusion period.

Data were analyzed according to a predefined statistical analysis plan which was deposited at the Data and Safety Monitoring Board before unblinding the study; post-hoc analyses were clearly stated as such. A 5%-significance level was used. All analyses were performed using STATA 13.1 (StataCorp LP, College Station, Texas, USA).

### Ethical considerations

This study was approved by the Committees on Biomedical Research Ethics (J.no. H-3–2010–087), the Danish Data Protection Board (J.no. 2009-41-4141), and the Danish Medicines Agency (J.no.2612-4356.EudraCT 2010-021979-85. Protocol 2009-323).

The study was registered September 22, 2012 at http://www.clinicaltrials.gov/ with registration number NCT01694108. The trial was supervised by the Good Clinical Practice Units of the Capital Region, Denmark and monitored by an independent Data and Safety Monitoring board. All methods were performed according to the Helsinki declaration and infants were included after oral and written informed consent from both parents.

### Data availability statement

Use of data will be confined to the study group, but potential collaborators or request for data can be submitted to the corresponding author.

## Results

During the inclusion period into the present study, a total of 601 infants were randomized at Copenhagen University Hospital, Hvidovre. Among these, 118 infants were included into this sub study and 483 infants were not. Of the 118 infants included, 56 were randomized to BCG and 62 to no intervention. Blood samples were collected from 114 infants at 4 days post randomization, 106 infants at 3 months, and 106 infants at 13 months (Fig. 1). The two groups had similar baseline characteristics (Table 2). There were only few drop-outs. However, the adherence was greater in the BCG group, the proportion of drop-outs being 4% (n = 2) in the BCG group vs. 16% (n = 10) in the control group (p = 0.04) (Table 2). Though it was not an exclusion criterion, none of the participating families had preterm children. Hence, our study population consisted of term (born between gestational weeks 37 + 0 and 41 + 6) children only. We have no information on infants whose parents declined the invitation to participate in this substudy. However, there were no differences in background characteristics, comparing infants included in the present study with infants randomized at Copenhagen University Hospital, Hvidovre in the inclusion period, but not included in the substudy (Supplementary Table B).

**Table 2:** Background characteristics at baseline for infants randomized to BCG or no intervention in the assessment of T and B cell subsets.

Enrollment	BCG	No BCG	p value
	n = 56	n = 62	
Age at randomization^a^			
*0–1 days*	32(57%)	41(66%)	0.32
*2–7 days*	24(43%)	21(34%)	
Male sex^a^	29 (52%)	32 (52%)	0.99
Gestational age (weeks)^b^	40 (38–41)	40 (38–41)	0.85
Weight (kg)^b^	3.5 (3.0–4.0)	3.5 (2.8–4.0)	0.21
Caesarean section^a^	7 (13%)	15 (24%)	0.10
Maternal BCG^a^	13 (24%)	9 (15%)	0.21
Parental etnicity different from Danish^a^	8 (14%)	11 (18%) [1]	0.58
Maternal smoking during pregnancy^a^	3(5%)	5 (8%) [1]	0.56
Level of maternal education^a^	[1]		0.56
*Basic schooling and non-theoretical education*	10 (18%)	14(23%)	
*Theoretical education incl BA level*	28 (51%)	25(41%)	
*Master level or more*	17 (31%)	22 (36%)	
Siblings^a^	19 (34%)	21 (34%)	0.99
Atopic predisposition^C^	39 (70%)	39(63%)	0.44
Lymphocytes (10^9/L) 4 days	5.5	5.3	0.43
Lymphocytes (10^9/L) 3 months	5.6	5.8	0.51
Lymphocytes (10^9/L) 13 months	5.7	6.0	0.40
Lost to follow-up^a^	2(4%)	10(16%)	0.04

^a^Number(Frequency) [missing].

^b^Median (10–90 percentiles) [missing].

^c^Atopic disposition is defined as at least one first degree relative with atopic disease. Atopic disease is defined as physician-diagnosed atopic eczema, asthma, allergic rhinoconjunctivitis or food allergy.

### Effect of BCG on T and B cell subsets at 4 days post-randomization

We found no difference between the BCG group and the control group on proportion of T or B lymphocyte subsets at 4 days post-randomization (Table 1).

### Immature cells and thymic output - naïve T cells

At 3 months, BCG-vaccinated infants had lower proportion of naïve CD4+ T cells than controls with GMR of 0.95, 95% CI (0.97–0.99), p = 0.01. No effects were found on RTEs.

### Memory cells: effector memory T cells (T_EM_) and late differentiated T cells

**Figure 4:**
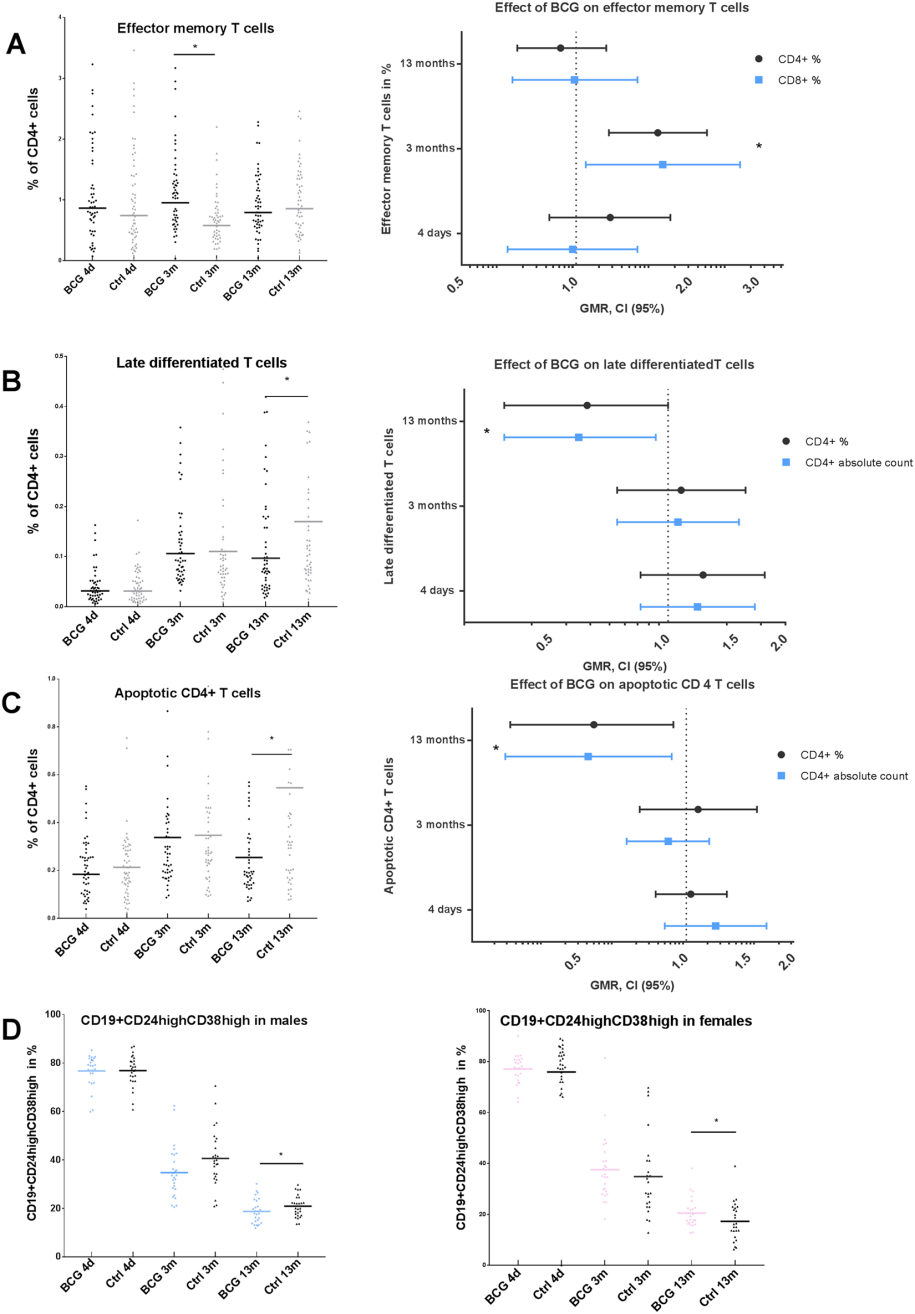
Effects of neonatal BCG on cell subsets reflecting T cell homeostasis and immune regulation. In the left column, dot plots illustrating % of CD 4+ effector memory cells, horizontal line representing the geometric mean (**A**), % of CD 4+ late differentiated cells (**B**), % of CD 4+ apoptotic cells (**C**), and CD19 + CD24^high^ + CD38^high^ cells (**D**) at 3 time points in BCG vaccinated infants and controls. The right column illustrates geometric mean ratio (GMR) of the cell phenotypes at all three time points (error bars depict 95% confidence intervals) except for **D** which shows % of CD19 + CD24^high^ + CD38^high^ in females.

At age 3 months, we found proportions of T_EM_ to be higher in the BCG group compared to controls for both CD4^+^ T cells (GMR 1.62, 95% CI (1.20–2.21), p = 0.002) (Fig. 4A) and CD8^+^ T cells (GMR 1.69, (1.06–2.70), p = 0.03) (Table 1). Furthermore, absolute count of CD4^+^ T_EM_ was higher in the BCG group (GMR 1.64, 95% CI (1.21–2.23), p = 0.002) (Table 1). This difference was not seen at 13 months.

At 13 months, BCG-vaccinated infants had fewer late differentiated CD4^+^ T cells compared to controls, both in proportions (GMR 0.62, 95% CI (0.38–1.00), p = 0.05) (Fig. 4B) and in absolute cell counts (GMR 0.59, 95% CI (0.38–0.93), p = 0.02) (Table 1).

### Apoptotic T cells

At 13 months, BCG-vaccinated infants had lower proportion of apoptotic CD4^+^ T cells than controls (GMR 0.55, 95% CI (0.32–0.92), p = 0.03) (Fig. 4C). The same was found for absolute counts of apoptotic CD4^+^ T (GMR 0.53, 95% CI (0.31–0.91), p = 0.02) **(**Table 1 **)**.

### Immune regulation

No overall effects were found on immune regulatory cells.

### Effect modification by sex

A sex differential effect of BCG was seen in CD19^+^CD24^high^CD38^high^ cells at the age of 13 months with a GMR 0.89, 95% CI (0.75–1.05) and 1.26, 95% CI (1.06–1.50) for boys and girls, respectively (p-value for interaction between BCG and sex = 0.005) (Fig. 4D). The same tendency was seen at 3 months: GMR for boys: 0.86, 95% CI (0.72–1.04) and for girls: GMR 1.12, 95% CI (0.93–1.35), p-value for interaction = 0.05. No sex differential effect was found in the other cell subsets (data not shown).

### Effect modification by time of vaccination

At all three time points, BCG-vaccinated infants randomized at the age of 0–1 days (early) had higher proportion of Tregs compared to controls, whereas BCG-vaccinated infants randomized at age 2–7 days (late) had lower proportion of Tregs compared to controls (Table 3). At 4 days post randomization, there was a difference in the effect of BCG between children randomized early or late in the proportion of CD19^+^CD24^high^CD38^high^ B cells; BCG was associated with a lower proportion in children vaccinated late. At 3 months of age, there was a difference in the effect of BCG between children randomized early or late in proportions of Th17 cells; BCG was associated with a higher proportion in children vaccinated late (Table 3).

**Table 3:** Effect modification by age of randomization in the assessment of the effect of neonatal BCG at birth on T and B cel subsets.

	0–1 days GM^a^		2–7 days GM		0–1 days GMR^b^ (95% CI)		2–7 days GMR (95% CI)		p value^c^
	BCG	No BCG	BCG	No BCG	BCG vs no BCG		BCG vs no BCG		
Cell subsets (%)	**4 days**								
	26^d^	35	19	20					
Tregs	9.1	7.5	7.5	8.9	1.22	(1.03–1.43)	0.84	(0.68–1.03)	**0.007**
TH 17 cells	0.6	0.6	0.9	0.6	1.01	(0.73–1.40)	1.42	(0.95–2.11)	0.19
TC 17 cells	1.5	1.8	1.7	1.6	0.85	(0.63–1.15)	1.08	(0.74–1.55)	0.33
CD19 + CD24^high^ + CD38^high^	77	60	76	81	1.02	(0.98–1.06)	0.94	(0.89–0.99)	**0.02**
	**3 months**								
	29	33	22	16					
Tregs	7.5	7.0	7.0	7.8	1.06	(0.96–1.18)	0.90	(0.79–1.02)	**0.04**
TH 17 cells	1.5	1.5	1.6	1.2	1.02	(0.84–1.24)	1.40	(1.11–1.79)	**0.04**
TC 17 cells	0.9	1.1	1.1	0.9	0.80	(0.61–1.05)	1.17	(0.84–1.63)	0.9
CD19 + CD24^high^ + CD38^high^	35	36	34	34	0.97	(0.81–1.15)	1.00	(0.81–1.23)	0.83
	**13 months**								
	29	34	22	15					
Tregs	7.9	7.2	7.5	8.2	1.10	(1.00–1.22)	0.91	(0.80–1.04)	**0.02**
TH 17 cells	2.3	2.2	2.5	2.1	1.05	(0.87–1.26)	1.15	(0.89–1.47)	0.56
TC 17 cells	0.8	0.8	0.8	0.7	1.00	(0.76–1.33)	1.15	(0.80–1.69)	0.54
CD19 + CD24^high^ + CD38^high^	20	18	18	19	1.11	(0.95–1.30)	0.95	(0.77–1.17)	0.24

The cell substes (given as proportions) included are cells affecting immune regulation (T regulatory cells (Tregs), TH 17 cells, TC 17 cells, and B cells with the phenotype of CD19 + CD24^high^ + CD38^high^) stratified by “age of randomization”.

^a^Geometric mean.

^b^Geometric mean ratio.

^c^p value for interaction.

^d^n number (%).

No effect modification by ‘age at randomization’ was found in the other cell subsets and no effect modification was found for maternal BCG vaccination (data not shown).

### Effect of BCG over time and in grouped analysis

No difference between randomizations groups within each cell subset was found over time (4 days, 3, and 13 months) and no difference between randomization groups within cells of “Immature T cells and thymic output”, “Immune regulation”, and “T cell homeostasis” was found in pooled analyses at 4 days, 3, and 13 months (data not shown).

## Discussion

Here we present data from the largest randomized study conducted to determine the possible effects of neonatal BCG vaccination on T and B lymphocyte subsets in healthy infants. A total of 118 healthy infants were randomized to neonatal BCG vaccination or to no intervention. No effect of BCG was found on the primary study outcome: proportions of any of the examined cell subsets 4 days post randomization. BCG was associated with higher proportions and absolute counts of both CD4^+^ T and CD8^+^ effector memory T cells at 3 months and reduced proportion and absolute counts of apoptotic and late differentiated cells at 13 months. In contrast, no overall difference was observed in naïve cells, RTEs, or cells affecting immune regulation. Finally, the association between BCG and cells of immune regulation (Tregs, Th17, and CD19^+^CD24^high^CD38^high^ B cells) differed by age at vaccination. A sex differential effect of BCG was found on CD19^+^CD24^high^CD38^high^ B cells. Thus, overall we found limited impact of neonatal BCG vaccination on T and B lymphocytes subsets; no clear pattern was identified that could theoretically explain non-specific beneficial effects of BCG.

Few previous studies investigated the effect of neonatal BCG in peripheral blood - *ex vivo*. In a study of 103 neonates activated T cells (CD4^+^CD25^+^) and natural Tregs (CD4^+^ CD25^+^FOXP3^+^) were measured at 4.5 months of age and compared between infants vaccinated at birth and BCG naïve infants, who were randomized to postponed BCG following blood sampling at 4.5 months^26^; they found no difference between groups. A tendency towards reduced proportions of Tregs (FoxP3 + CD45RO + CD4+) assessed at age 2–3 months following neonatal BCG (mean BCG vs. no BCG was 9.1 vs. 11.6, p = 0.07) was however demonstrated in a study from the Philippines^27^. In contrast, in whole blood cultures, after antigen-specific stimulation, several effects of BCG have been described in infants; there amongst the induction of both a Th1 and a Th2 response^26,28,29^, a Th17 response^26,29^, mycobacterial-specific polyfunctional CD4^+^ T cells (producing a combination of IFN-γ, interleukin (IL)-2, and tumor necrosis factor (TNF)-a)^30–34^, and Tregs^26,35,36^.

Our findings of no effect on RTEs, which are T cells recently generated in the thymus^37^, are in line with our findings with respect to BCG and thymic size; within the same study population BCG did not affect thymic size measured as thymic index by ultrasound^38^. This suggests that any effect of BCG is not mediated by affecting thymic size or thymic output.

In memory cell subsets, BCG vaccination resulted in higher proportion of CD4^+^ T_EM_ cells at 3 months of age. Likewise, increases in both absolute counts of CD4^+^ T_EM_ cells and in proportions of CD8^+^ T_EM_ cells were seen. We speculate that these changes may represent induced T cell memory and hence specific effects of the vaccine. However, functional assays to determine antigen-specific alterations in T and B cell subpopulations were not performed, but may have provided important information. CD4^+^ T_EM_ cells have been described to migrate to inflamed tissues following antigen exposure, where they display immediate effector functions. In contrast, central memory cells (T_CM_) home to T cell areas of secondary lymphoid organs, they are long-lived and show limited effector functions, but differentiate to effector cells upon antigen exposure^39^. Since CCR7^−^ effector T cells (T_EM_) have been associated with persistent activation of T cells in e.g. chronic viral infections where the antigen is not cleared^40–42^ and in children with active TB^43^, Soares *et al*. suggest that their similar finding of T_EM_ cells (CD45RA^−^ CD27^+^CCR7^−^) to be the dominant phenotype 10 weeks following neonatal BCG, may reflect the persistence of BCG^31^. Our findings of an increase at 3 months of T_EM_ with an identical phenotype corroborate these results. Furthermore, in our cohort, in case of suppurative lymphadenitis as an adverse reaction to BCG, the mean time for onset was 87 days (range 25–200) after vaccination, supporting an BCG induced immunologic reaction 3 months post vaccination^44^. This persistence of BCG was hypothesized to drive differentiation predominantly into effector cells and prevent the differentiation into central memory cells and long lived memory^31^.

A tendency towards higher proportion of CD4^+^ T_CM_ cells at 13 months was found in the BCG group compared to controls. It could be speculated that this shift from an increase in T_EM_ at 3 months to an increase in T_CM_ at 13 months, may indicate the conversion of effector memory to central memory cells and thus potentially long lived memory. The function and differentiation between memory T cells may, however, be even more complex, underlined by previous findings in infants of BCG-specific CD4^+^ T cells with a central memory phenotype but with functional features of effector memory cells >10 weeks post vaccination^34^. Our study included no functional assays, and we are not able to elaborate on the functional features of the T memory cells. At 13 months, fewer late differentiated cells and apoptotic cells, measured as both proportions and absolute counts, were found in the BCG group compared to the control group. Of note, few cells of both late differentiated cells and apoptotic cells were found, and random variation may have affected the results.

No overall effects were found on any immune regulatory cell subset, and as such we found no clear indication of altered proportions or absolute counts of cells immune regulation after BCG vaccination at birth. However, in analyses of effect modification our results suggest that effects of BCG on regulatory cells (Tregs, CD19^+^CD24^high^CD38^high^ cells, and Th17 cells) differ, depending on age of vaccination. In parallel, a similar finding was done in another immunological substudy nested within the Danish Calmette Study, which assessed the effect of BCG on antibody response following routine vaccination^45^. Here, BCG was associated with higher levels of antibodies if given at day 2–7 versus at day 0–1^45^. In the present study, BCG induced an increase in the proportions of Tregs following early (0–1 days) BCG vaccination, whereas the opposite effect was seen after late (2–7 days) BCG. Hence, age of BCG vaccination may be important in the immune regulating capacity of BCG, and we speculate that an increase in proportions of Tregs, in part, may explain lower antibody production. The mechanism may be the ability of Tregs to suppress development of long-lived plasma cells, as recently described in mice^46^. Since regulatory T and B cells are thought to play an important role in both vaccine immunogenicity in early life^47^ and in development of autoimmune disease^48^, such time differential effects may be pursued in future studies.

Timing of sampling is important. Sampling 4 days post randomization was chosen to asses early effect of BCG, since a rapid beneficial effect of BCG on was seen on all-cause mortality in newborns within few days after vaccination in Guinea Bissau^8^. To assess the effect of BCG without any potential impact by other vaccines the 3-month-blood sample was scheduled before the first dose of the pentavalent vaccine (DiTeKiPol/Act-Hib) and the pneumococcal conjugate vaccine (Prevenar 13), which are recommended at 3, 5 and 12 months as part of the standard vaccination program in Denmark. The 13-month-blood sample was scheduled 4 weeks after the last dose DiTeKiPol/Act-Hib and Prevenar 13, since DiTeKiPol has been proposed to have detrimental NSEs^49^ on infants heath. Based on previous findings of a peak response to neonatal BCG at 6–10 weeks of age, where after it gradually waned^50^, and the fact that adaptive immunity takes weeks to develop, it could be argued, that 4 days post vaccination may be too premature to assess changes within the adaptive immune system. In contrast, it is not possible to rule out that 3 months may be too late to capture the peak in specific response. However, a tendency towards reduced proportions of Tregs with the phenotype FoxP3 + CD45RO + CD4+ as well as the induction of a Th 1 immune response were demonstrated after sampling at age 2–3 months following neonatal BCG^27^.

We observed a sex differential effect of BCG on CD19^+^CD24^high^CD38^high^ cells, with higher proportions in females and lower proportions in males compared to controls at 13 months. The proportions of CD19^+^CD24^high^CD38^high^ cells in this study, however, were high (76% at 4 days post randomization), and it is likely that this phenotype includes other cells than IL-10 producing cells. Our results are in line with findings of sex differences in immune function and response to vaccination^51,52^ including BCG^8,25^, with females typically developing higher antibody responses to vaccines^53^.

The hypothesis that live vaccines have stronger beneficial NSEs in children born of mothers who were primed with the same vaccine was first invoked after finding that infants vaccinated with measles vaccine in the presence of maternal measles antibodies had a lower mortality than infants vaccinated in the presence of no maternal antibodies^54^. Maternal BCG status was therefore assessed within the Danish Calmette Study, and as hypothesized, a potential beneficial positive NSE of BCG was found on infectious disease episodes^55^ and hospitalizations for infectious diseases among children whose mothers were BCG-vaccinated, whereas no effect was seen in children of BCG-unvaccinated mothers (Stensballe *et al*., “BCG vaccination at birth and rate of hospitalization for infection until 15 months of age in Denmark. A randomized clinical multicenter trial”, submitted for publication). We found no effect modification by maternal BCG on T and B cell subsets, however only 20% of the mothers were BCG vaccinated, providing low power.

In a clinical perspective, no effect of BCG was found on the primary outcome of the Danish Calmette Study^16^: overall hospitalizations at 15 months of age (hazard ratio comparing BCG vs. no BCG of 1.05, 95% CI 0.93–1.18)^15^. Also, BCG did not affect parent-reported infections (incidence rate ratio comparing BCG vs. no BCG of 0.87, 95% CI 0.72–1.05 from 0–3 months and of 1.02, 95% CI: 0.97–1.07 from 3–13 months)^55^ or recurrent wheeze in the first year of life (relative risk comparing BCG vs. no BCG of 1.07, 95% CI 0.89–1.28)^56^. Furthermore, no effect of BCG on overall antibody response to the routine vaccines against DiTeKiPol/Act-Hib and Prevenar 13 at 13 months of age was found^45^; nor on *in vitro* cytokine responses to specific and non-specific stimulation (Nissen *et al*., Bacillus Calmette-Guérin vaccination at birth and *in vitro* cytokine responses to specific and non-specific stimulation. A randomized clinical trial, submitted for publication). However, BCG was found to protect against atopic dermatitis among newborns with atopic predisposition at 13 months (Thøstesen *et al*., Neonatal BCG-vaccination and atopic dermatitis before 13 months of age. A randomised clinical trial, submitted for publication). Taken together these findings suggest limited NSEs in a Danish cohort of infants. This is in agreement with the limited overall impact of neonatal BCG on T cells and T cell function within the first year of life in Danish children.

Strengths of this study are the randomized and prospective design with an overall follow-up rate of 90% and few drop-outs. The lack of intracellular staining of FOX-P3 (Forkhead Box P3) in the assessment of Tregs and the lack of IL-10 staining in the assessment of CD19^+^CD24^high^CD38^high^ cells, which was not logistically feasible, represents weaknesses of this study. A limitation is that the recruitment was based on convenience sampling with the risk of selection bias. Functional assays may have provided important information. We did not adjust for multiple testing which imposes the risk of a type 1 error. However, due to the explorative nature of this study and based on adherence to a predefined statistical analysis plan, we chose to report data without adjusting for multiple testing and let the reader interpret the analysis as exploratory. However, the reader should keep the risk of type 1 error in mind^57^.

In conclusion, limited impact of BCG vaccination at birth on lymphocyte subsets was found in healthy infants in a high-income setting within the first 13 months of life. Since no marked effect of BCG was found on clinical outcomes in our setting, it is not possible to rule out that BCG may affect lymphocyte subsets and lead to NSEs in other settings.

## Electronic supplementary material

Supplementary Table A and B
