# Supplementary material for: Effects of Bacillus Calmette-Guérin (BCG) vaccination at birth on T and B lymphocyte subsets: Results from a clinical randomized trial
Source: Sci Rep. 2017 Sep 29;7:12398. doi: 10.1038/s41598-017-11601-6 (PMC5622034; doi:10.1038/s41598-017-11601-6)
Supplement: Supplementary file 1 — Supplementary Table A and B [file 41598_2017_11601_MOESM1_ESM.pdf]

# Title: Effects of Bacillus Calmette-Guérin (BCG) vaccination at birth on T and B lymphocyte subsets. Results from a clinical randomized trial.

## Author list:

Nina Marie Birk[1]#, Thomas Nørrelykke Nissen[1] , Jesper Kjærgaard[2] , Hans Jacob Hartling[3] , Lisbeth Marianne Thøstesen[4] , Poul-Erik Kofoed[4] , Lone Graff Stensballe[2] , Andreas Andersen[5] , Ole Pryds1 , Mihai G. Netea[6] , Christine Stabell Benn[5] , Susanne Dam Nielsen[3] , Dorthe Lisbeth Jeppesen[1] .

1. Department of Pediatrics, Copenhagen University Hospital, Hvidovre, Kettegårds Allé 30, 2650 Hvidovre, 13 Denmark.

2. The Department of Pediatrics and Adolescent Medicine, Copenhagen University Hospital, Rigshospitalet, Blegdamsvej 9, 2100 Copenhagen, Denmark.

3. Department of infectious diseases, Copenhagen University Hospital, Rigshospitalet, Blegdamsvej 9, 2100, Copenhagen Denmark.

4. Department of Pediatrics, Kolding Hospital, Sygehusvej 24, 6000 Kolding, Denmark.

5. Research Center for Vitamins and Vaccines (CVIVA), Bandim Health Project, Statens Serum Institut, Artillerivej 5, 2300 Copenhagen,Denmark.

6. Department of Internal Medicine and Radboud Center for Infectious Diseases, Radboud University Medical 22 Center, Geert Grooteplein 8, 6525 GA, Nijmegen, The Netherlands

Corresponding author: Birk, Nina Marie, MD. Department of Pediatrics, 460, Copenhagen University Hospital, 25 Hvidovre, Kettegaard Allé 30, DK-2650 Hvidovre, Denmark. Telephone: 0045 27202037, email: ninabirk@dadlnet.dk

**Supplementary table A. Geometric means (GM) of lymphocyte subsets of both proportions and absolute counts assessed by flow cytometry in BCG vaccinated infants and their controls at three time points.** [a] Geometric mean . [b] (cells/µL)

| | **4 days** BCG (n=53) vs no BCG (n=61) | | | | **3 months** BCG (n=53) vs no BCG (n=53) | | | | **13 months** BCG (n=54) vs no BCG (n=52) | | | |
|---|---|---|---|---|---|---|---|---|---|---|---|---|
| | GM[a] ***proportion (%)*** | | GM ***cell count***[b] | | GM ***proportion (%)*** | | GM ***cell count*** | | GM ***proportion (%)*** | | GM ***cell count*** | |
| | BCG | No BCG | BCG | No BCG | BCG(n) | No BCG(n) | BCG | No BCG | BCG | No BCG | BCG | No BCG |
| **CD 4+ T cell subsets** | | | | | | | | | | | | |
| *CD4+ T cells* | | | 1815 | 1535 | | | 2421 | 2403 | | | 2215 | 2358 |
| Naive cells | 93 | 93 | 1647 | 1397 | 92 | 93 | 2221 | 2242 | 88 | 89 | 1987 | 2112 |
| Recent thymic emmigrants | 59 | 57 | 1039 | 858 | 62 | 60 | 1489 | 1437 | 62 | 62 | 1393 | 1453 |
| Tregs | 8.4 | 8.0 | 154 | 121 | 7.3 | 7.3 | 162 | 174 | 7.7 | 7.5 | 173 | 176 |
| Th 17 cells | 0.7 | 0.6 | 12 | 8.9 | 1.6 | 1.3 | 35 | 33 | 2.3 | 2.2 | 52 | 53 |
| Central memory cells | 4.4 | 3.8 | 74 | 58 | 4.5 | 3.7 | 110 | 90 | 8.2 | 6.1 | 182 | 145 |
| Effector memory cells | 0.9 | 0.7 | 14 | 11 | 1.0 | 0.6 | 24 | 15 | 0.8 | 0.9 | 18 | 20 |
| Late differentiated cells | 0.03 | 0.03 | 0.6 | 0.5 | 0.1 | 0.1 | 2.9 | 2.7 | 0.1 | 0.2 | 2.4 | 4.0 |
| Chronic activated cells | 1.0 | 0.8 | 18 | 13 | 1.0 | 1.2 | 23 | 27 | 1.1 | 1.3 | 25 | 31 |
| Apoptotic cells | 0.2 | 0.2 | 3.5 | 2.9 | 0.3 | 0.3 | 7.2 | 8 | 0.3 | 0.5 | 6.8 | 13 |
| **CD8+ T cell subsets** | | | | | | | | | | | | |
| *CD8+ T cells* | | | 502 | 464 | | | 687 | 761 | | | 789 | 884 |
| Naive cells | 94 | 95 | 458 | 434 | 84 | 86 | 576 | 652 | 80 | 76 | 631 | 678 |
| Recent thymic emmigrants | 96 | 97 | 469 | 443 | 90 | 90 | 618 | 682 | 86 | 83 | 678 | 748 |
| Tc 17 cells | 1.6 | 1.7 | 7.2 | 7.8 | 0.9 | 1.0 | 5.8 | 7.9 | 0.8 | 0.8 | 6.7 | 7.2 |
| Central memory cells | 0.8 | 0.8 | 3.7 | 3.8 | 0.9 | 0.8 | 6.0 | 6.0 | 1.4 | 1.1 | 11 | 10 |
| Effector memory cells | 0.3 | 0.3 | 1.2 | 1.3 | 1.0 | 0.6 | 6.8 | 4.8 | 1.3 | 1.4 | 10 | 12 |
| Late differentiated cells | 0.9 | 1.0 | 4.3 | 4.8 | 0.8 | 0.8 | 5.2 | 6.2 | 0.9 | 1.5 | 6.9 | 13 |
| Chronic activated cells | 1.6 | 1.5 | 8.5 | 6.8 | 2.5 | 3.1 | 17 | 24 | 2.6 | 2.9 | 20 | 26 |
| Apoptotic cells | 4.0 | 4.0 | 20 | 18 | 6 | 6.4 | 41 | 49 | 11 | 14 | 91 | 120 |
| **CD 19+ B cell subsets** | | | | | | | | | | | | |
| *CD19+ B cells* | | | 391 | 365 | | | 1230 | 1213 | | | 1342 | 1340 |
| CD19+CD24$^{high}$+CD38$^{high}$ | 77 | 78 | 302 | 279 | 35 | 36 | 428 | 438 | 19 | 18 | 251 | 242 |

**Supplementary table B. Background characteristics for infants randomized to BCG or no intervention in The Danish Calmette Study. Comparing infants included in the substudy on T and B cell subsets with infants who were randomized at Copenhagen University Hospital, Hvidovre in the inclusion period, but who were not included into the substudy.**

| | **Substudy on T and B cells** | **Randomized at HvH** | p value |
|---|---|---|---|
| **Enrollment** | **n=118** | **n=483** | |
| Age at randomization[a] | | | |
| *0-1 days* | 73 (62%) | 250 (52%) | 0.05 |
| *2-7 days* | 45 (38%) | 233 (48%) | |
| Male sex[a] | 61 (52 %) | 237 (49 %) | 0.30 |
| Gestational age (weeks)[b] | 40 (38-41) | 40 (38-41) | 0.33 |
| Weight (Kg)[b] | 3.5 (2.9-4.0) | 3.5 (2.8-4.0) | 0.54 |
| Caesarean section[a] | 22 (19 %) | 121 (25 %) | 0.14 |
| Maternal BCG[a] | 22 (19%) | 93 (20%) [6] | 0.87 |
| Parental etnicity different from Danish[a] | 19 (16%)[1] | 116 (24%) | 0.07 |
| Maternal smoking during pregnancy[a] | 8 (7%) [1] | 53 (11%) | 0.17 |
| Level of maternal education[a] | [1] | | 0.56 |
| *Basic schooling and non-theoretical education* | 25 (21%) | 112 (23%) | |
| *Theoretical education incl BA level* | 53 (45%) | 223 (46%) | |
| *Master level or more* | 39 (34%) | 148 (31%) | |
| Siblings[a] | 40 (34%) | 232 (39%) | 0.24 |
| Atopic predisposition[c] | 78 (66%) | 289 (60%) | 0.21 |

[a] N number (Frequency) [not available],

[b]Median (10-90 percentiles)[not available]

[c] Atopic disposition is defined as at least one first degree relative with atopic disease. Atopic disease is defined as physician-diagnosed atopic eczema, asthma, allergic rhino-conjunctivitis or food allergy
